# Kidney Transplant Recipients Develop Nasal Mucosal Antibodies to SARS‐CoV‐2 With ACE2‐Inhibiting Activity Following mRNA Vaccination

**DOI:** 10.1111/tid.70230

**Published:** 2026-05-07

**Authors:** Vera J. C. H. Koomen, Christa E. van der Gaast‐deJongh, Fred van Opzeeland, Ria Philipsen, Marije C. Baas, A. Lianne Messchendorp, Frederike J. Bemelman, Marcia M. L. Kho, Carla C. Baan, Debbie van Baarle, Jan‐Stephan F. Sanders, Rob van Binnendijk, Gerco den Hartog, Ron T. Gansevoort, Renate G. van der Molen, Luuk B. Hilbrands, Dimitri A. Diavatopoulos

**Affiliations:** ^1^ Department of Nephrology Radboud University Medical Center Nijmegen Nijmegen the Netherlands; ^2^ Department of Laboratory Medicine Laboratory of Medical Immunology Radboud University Medical Center Nijmegen Nijmegen the Netherlands; ^3^ Division of Nephrology Department of Internal Medicine University of Groningen University Medical Center Groningen Groningen the Netherlands; ^4^ Department of Medical Microbiology and Infection Prevention University of Groningen University Medical Center Groningen Groningen the Netherlands; ^5^ Renal Transplant Unit Amsterdam UMC University of Amsterdam Amsterdam the Netherlands; ^6^ Department of Internal Medicine Nephrology and Transplantation Erasmus MC Transplant Institute Erasmus Medical Center Rotterdam the Netherlands; ^7^ Center For Infectious Disease Control National Institute for Public Health and the Environment De Bilt the Netherlands

**Keywords:** ACE2 inhibition, kidney transplant recipients, mucosal antibodies, neutralizing antibodies, SARS‐CoV‐2, vaccination

## Abstract

**Background:**

Less than 60% of kidney transplant recipients (KTRs) become IgG seropositive for the viral spike (S) antigen following two COVID‐19 vaccine doses, with a third dose increasing these numbers further. Mucosal antibodies play a critical role in protection against SARS‐CoV‐2 (re)infection; however, the presence and functionality of these antibodies after vaccination have not been well‐studied in high‐risk groups with reduced vaccine immunogenicity.

**Methods:**

We assessed the concentration of ancestral S‐specific antibodies in the nasal mucosa and in serum as well as their capacity to neutralize binding of the receptor‐binding domain (RBD) to the ACE2 receptor in KTRs after two, three, and/or four vaccinations, compared with an age‐matched control group after two vaccine doses.

**Results:**

KTRs showed an increase in nasal and serum S‐specific IgG and S‐specific IgA geometric mean concentrations (GMCs) as well as ACE2 binding inhibition at 28 days after both two‐dose and additional vaccinations compared to baseline, although postvaccination GMCs remained lower than in the control group. We observed a high correlation between nasal and serum antibody levels (*r*_S_ > 0.63) and ACE2 inhibiting capacity (*r*_S_ > 0.65). Furthermore, nasal S‐specific IgG, and to a lesser extent IgA, was correlated with nasal ACE2 binding inhibition (*r*_S_ > 0.64 and 0.5).

**Conclusions:**

KTRs develop nasal mucosal antibodies, which are able to inhibit binding to ACE2 after COVID‐19 vaccination, but at a lower concentration than controls. Mucosal sampling provides an accessible and minimally invasive addition to measuring serum antibodies for monitoring the response to vaccination at a population level.

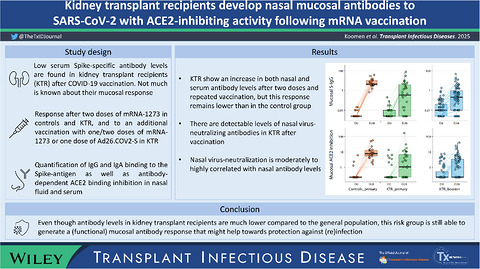

## Graphical abstract

Kidney transplant recipients develop nasal mucosal antibodies after systemic COVID‐19 vaccination, which are able to inhibit binding of SARS‐CoV‐2 to the ACE2 receptor, but at a lower concentration than the general population.

**Abbreviations**
ACE2: angiotensin‐converting enzyme 2
BAU: binding antibody units
COVID‐19: coronavirus disease 2019
GMC: geometric mean concentration
IAU: inhibiting arbitrary units
KTRs: kidney transplant recipients
LLOD: lower limit of detection
MIA: multiplex immunoassay
MLF: mucosal lining fluid
MMF: mycophenolate mofetil
N: nucleocapsid
S: spike
SARS‐CoV‐2: severe acute respiratory syndrome coronavirus‐2

## 1 Introduction

After the start of the COVID‐19 pandemic, vaccines against SARS‐CoV‐2 were developed and licensed with incredible speed. Due to limited availability, vaccination was initially prioritized for high‐risk groups such as healthcare workers, older people, and immunocompromised patients. The latter includes patients with severely reduced kidney function and kidney transplant recipients (KTRs), who were found to be at three‐ to four‐fold higher risk of severe infection and mortality due to COVID‐19 [1]. Repeated vaccine administration has subsequently been offered and is now widely implemented in booster vaccination programs, particularly for high‐risk groups.

We and others have previously shown reduced immunogenicity of mRNA vaccination against COVID‐19 in KTRs, with less than 60% of KTRs showing IgG seropositivity for the viral spike (S) antigen following two primary vaccine doses [2, 3, 4, 5, 6]. The use of immunosuppressive drugs, in particular mycophenolate mofetil (MMF), is a major risk factor for a weak antibody response to vaccination [2, 7, 8, 9]. Although administration of additional doses increases the proportion of seropositive KTRs, serum anti‐S IgG levels remain lower than in healthy controls [10, 11, 12, 13].

Whilst previous studies have primarily focused on systemic immune responses to vaccination in KTRs, the mucosal response has thus far not been investigated in this patient cohort. Mucosal antibodies play an important role in protection against COVID‐19 by preventing (re)infection with SARS‐CoV‐2, or in case infection cannot be prevented, by restricting viral replication and spread to the lower respiratory tract and beyond [14, 15, 16]. The analysis of nasal mucosal antibodies may offer a suitable supplement to serum antibodies, as sampling via nasosorption is less invasive than the drawing of blood samples [17]. Although several studies have shown that SARS‐CoV‐2 vaccination generates a mucosal antibody response against the S‐protein [16, 18, 19], the presence of antibodies in the nose after intramuscular vaccination is thought to be derived from serum and not due to local production, in contrast to nasal antibodies that are detected following infection [20, 21]. We have previously shown that the mode of primary exposure, that is, vaccination or infection, influences mucosal serotype distribution, and the relative contribution of IgG and IgA to virus neutralization [22].

In this study, we analyzed nasal mucosal and serum antibody responses to both primary doses and repeated COVID‐19 vaccination in KTRs and compared these to controls after primary vaccination. We quantified IgG and IgA binding to S‐antigen as well as antibody‐dependent ACE2 binding inhibition, and investigated the relationship between nasal and serum antibodies. Our study provides new insights into the mucosal antibody response to primary and repeated COVID‐19 vaccination in KTRs.

## 2 Methods

### 2.1 Cohort Description

This is an exploratory, immunological sub‐study of the RECOVAC Immune Response (RECOVAC‐IR) and the RECOVAC Repeated Vaccination (RECOVAC‐RV) studies (Figure S1).

The RECOVAC‐IR study was a prospective, controlled multicenter study designed to investigate the immunogenicity and safety of COVID‐19 vaccination in patients with chronic kidney disease, dialysis patients, and KTRs, compared with healthy controls. Participants received two COVID‐19 vaccinations between February 24 and April 8, 2021 (ClinicalTrials.gov identifier: NCT04741386). For the present sub‐study, we used an online randomization tool to select half of the participants enrolled at Radboud University Medical Center (Radboudumc), Nijmegen: 23 controls and 36 KTRs (KTR‐IR). All participants received two doses of spikevax (mRNA‐1273; Moderna Biotech Spain, S.L.), administered 28 days apart. Nasal mucosal lining fluid (MLF) and serum samples were obtained at baseline (D0) and 28 days (D28) after the second dose.

All participants included in the RECOVAC‐IR study were seronegative at baseline, defined as serum S‐specific IgG < 10 BAU/mL. None of the participants included in this analysis had a history of a positive PCR SARS‐CoV‐2 test or detectable serum antibody levels against the SARS‐CoV‐2 nucleocapsid (N) protein at either baseline or D28.

In addition, we included participants enrolled at Radboudumc in the RECOVAC‐RV study, a prospective multi‐center study investigating the immunogenicity of repeated COVID‐19 vaccination in KTRs who remained seronegative (serum S‐specific IgG < 10 BAU/mL) 28 days after two vaccinations with mRNA‐1273 (ClinicalTrails.gov identifier: NCT05030974). Participants received a third or fourth dose of COVID‐19 vaccination between November 2 and November 11, 2021 with a single or double dose of mRNA‐1273, or a heterologous dose of Ad26.COV2‐S. For the current analysis, all 76 RECOVAC‐RV participants from Radboudumc were included: 25 received a single dose of mRNA‐1273 vaccine, 25 received a double dose of mRNA‐1273, and 26 received a single dose of Ad26.COV2‐S. As no significant differences in immunogenicity were observed between the three vaccination arms in previous analyses, these subgroups were pooled for this study (KTR‐RV) [10]. MLF and serum were obtained at baseline (D0) and 28 days after repeated vaccination (D28). None of the RECOVAC‐RV participants included in this analysis had a history of a positive PCR SARS‐CoV‐2 test or detectable serum antibodies against the SARS‐CoV‐2 N‐antigen at either baseline or D28.

Both the RECOVAC‐IR and RECOVAC‐RV studies were approved by the local ethics committee, and all participants provided written informed consent before participating. Details on the study design and primary endpoints from both studies have been published previously [2, 10, 23].

### 2.2 Sample Analysis

#### 2.2.1 MLF Sampling

The method of MLF sampling for antibody analysis has previously been described in detail [17]. In short, a Nasosorption FX·i nasal sampling device (Hunt Developments, UK) was gently inserted into the nose for 60 s. Following nasosorption, the sampling devices were frozen immediately and stored at −20°C until further testing. Following thawing, the synthetic adsorptive matrix (SAM) was removed using sterile forceps and placed in a spin‐X filter Eppendorf tube with 300 µL elution buffer (PBS/1% BSA) for 10 min, followed by centrifugation at 16 000 × *g* for 10 min. The eluted MLF was incubated for 1 h at 56°C before storage at −20°C and used in immunological assays.

#### 2.2.2 Antibody Quantification

The quantification of nasal mucosal and serum antibodies with bead‐based multiplex immunoassay (MIA) has been described previously [17]. Briefly, Wuhan‐Hu‐1 trimeric S (D614G mutant; ExcellGene), Wuhan‐Hu‐1 N‐His recombinant (Sino Biological), and Wuhan‐Hu‐1 RBD (ExcellGene) antigens were conjugated to Luminex MagPlex beads 02, 24, and 60, respectively. The beads were incubated for 45 min at room temperature with MLF (1:5) or serum (1:500) samples, washed, and then incubated in a 1:200 dilution with either phycoerythrin (PE)‐conjugated goat anti‐human IgG or IgA (Southern Biotech) for 20 min. Samples were measured on the Luminex Flexmap 3D System with xPONENT software. MLF IgG and IgA, and serum IgA were measured at the Radboudumc, while serum IgG was previously measured at the National Institute for Public Health and the Environment (Bilthoven, the Netherlands) with the same protocol and published [2].

Mean fluorescence intensity (MFI) values were converted to binding antibody units (BAU) using an 11‐point serial dilution of in‐house reference serum, calibrated to the World Health Organization's International Standard [24]. Samples were interpolated to the IgA or IgG standard curve with a log 5‐parameter logistic regression and log–log axis transformation using BioPlex Manager 6.2 software (Bio‐Rad Laboratories), and subsequently exported to RStudio. Samples with an interpolated BAU value below the lower limit of detection (< LLOD) of their respective plate were manually assigned half the value of the lowest LLOD per sample and analyte type. Samples with a value above the upper limit of detection were re‐measured in a higher dilution. When an in‐range sample was measured more than once, the mean of the values was used for analysis.

#### 2.2.3 ACE2 Binding Inhibition Assay

The capacity of nasal and serum antibodies to block binding of ACE2 to the S‐antigen was analyzed using an in‐house bead‐based ACE2 binding inhibition assay as a surrogate measure of neutralizing activity, as previously described [22]. In short, the same antigen‐conjugated magnetic beads from the MIA were used and incubated for 30 min with MLF (undiluted) or serum (1:200) samples at room temperature. Subsequently, human recombinant (His‐Tag) biotinylated ACE2 protein dilution (Sino Biological; corresponding to 0.25 mg/mL) was added for 30 min at room temperature. After washing, the samples were incubated with a 1:50 dilution of Streptavidin R‐PE conjugate (SAPE; Fisher Scientific) for 20 min. An 11‐point dilution of an in‐house standard was added to each plate and the level of inhibiting arbitrary units per milliliter (IAU/mL) was calculated by the same method as the MIA. Samples with an interpolated IAU value below the LLOD were manually assigned a value half of the lowest LLOD per sample type.

#### 2.2.4 Secretory Nature of Mucosal IgA

To assess whether IgA in MLF versus serum is predominantly monomeric or multimeric, both independent and dependent of antigen specificity, additional exploratory experiments were performed on a small subset of nasal MLF and serum samples from each study group. Briefly, anti‐human IgA antibody (for antigen‐independent analysis; Biolegend) and Wuhan‐Hu‐1 trimeric S‐antigen (for antigen‐specific analysis; ExcellGene) were conjugated to Luminex MagPlex beads 09 and 02, respectively. The beads were incubated for 45 min at room temperature with MLF (undiluted) or serum (1:500) samples, washed, and then incubated with either a 1:50 dilution of anti‐Ig J‐chain PE‐conjugated antibody (Santa Cruz Biotechnology) or with a 1:200 dilution of PE‐conjugated goat anti‐human IgA (Southern Biotech) for 20 min.

### 2.3 Statistical Analysis

All samples were randomized over the plates before measurement, with paired timepoints from the same subject on the same plate. Geometric mean concentrations (GMCs) were calculated for each group at each time point. Differences between the timepoints were analyzed with the two‐tailed Wilcoxon signed‐rank test, differences between the groups were analyzed with the two‐tailed Mann–Whitney *U*, thereby only comparing groups within the same clinical study. Correlations were calculated with a Spearman rank correlation. Correlation analysis between sample type, antibody type, or assays was only performed on the samples that had in‐range values for both parameters. The significance threshold was set at a two‐sided *p*‐value < 0.05. All analyses were performed in RStudio version 2022.02.1, using R software version 4.1.3.

## 3 Results

### 3.1 Cohort Description

This study included participants from the RECOVAC‐IR and RECOVAC‐RV studies, see Table 1. Among RECOVAC‐IR participants, the median age of controls (*N* = 23) and KTR‐IR (*N* = 36) was 55 and 59 years, respectively, with 52% and 36% female participants. Within the KTR‐IR group, different immunosuppressive regimens were used, with MMF prescribed in 44% of participants. The KTR‐RV group (*N* = 76) had a median age of 61 years, included 41% female participants, and 63% were receiving MMF. No significant differences were observed with respect to age, biological sex, or other general clinical characteristics between controls and KTRs, apart from variables related to kidney transplantation. For further participant characteristics of the KTR‐RV subgroups, see Table S1.

**TABLE 1:** Participant characteristics.

	Controls‐IR (*N* = 23)	KTR‐IR (*N* = 36)	KTR‐RV (*N* = 76)
Age (y), median (range)	55 (23–73)	61 (46–77)	60 (28–77)
Female, *n* (%)	12 (52.2)	13 (36.1)	31 (40.8)
Caucasian, *n* (%)	22 (95.7)	36 (100)	74 (97.4)
BMI (kg/m^2^), median (range)	25.7 (20.0–34.5)	26.9 (20.3–36.9)	25.0 (17.4–45.6)
Years since transplant, median (range)	NA	10 (1–34)	5 (0–35)
Vaccine platform and dosing, *n* (%)			
One dose of mRNA‐1273	23 (100)	36 (100)	25 (32.9)
Two doses of mRNA‐1273	NA	NA	25 (32.9)
One dose of Ad26.COV2‐S	NA	NA	26 (34.2)
Immunosuppressive medication, *n* (%)			
Mycophenolate mofetil	NA	16 (44.4)	48 (63.2)
Azathioprine	NA	4 (11.1)	5 (6.6)
Calcineurin inhibitors	NA	27 (75.0)	59 (77.6)
mTOR inhibitors	NA	3 (8.3)	5 (6.6)
Steroids	NA	29 (80.6)	48 (63.2)
Three‐drug regimen	NA	7 (19.4)	14 (18.4)
Comorbidities, *n* (%)			
Hypertension	5 (21.7)	32 (88.9)	67 (88.2)
Diabetes mellitus	2 (8.7)	7 (19.4)	26 (34.2)
History of coronary artery disease	0 (0.0)	5 (13.9)	8 (10.5)
Heart failure	0 (0.0)	0 (0.0)	1 (1.3)
Chronic lung disease	0 (0.0)	1 (2.8)	10 (13.2)
History of malignancy	0 (0.0)	5 (13.9)	16 (21.1)
Autoimmune disease	0 (0.0)	3 (8.3)	3 (3.9)

### 3.2 Nasal Mucosal Antibody Levels Correlate With Serum Antibody Levels

**FIGURE 1:**
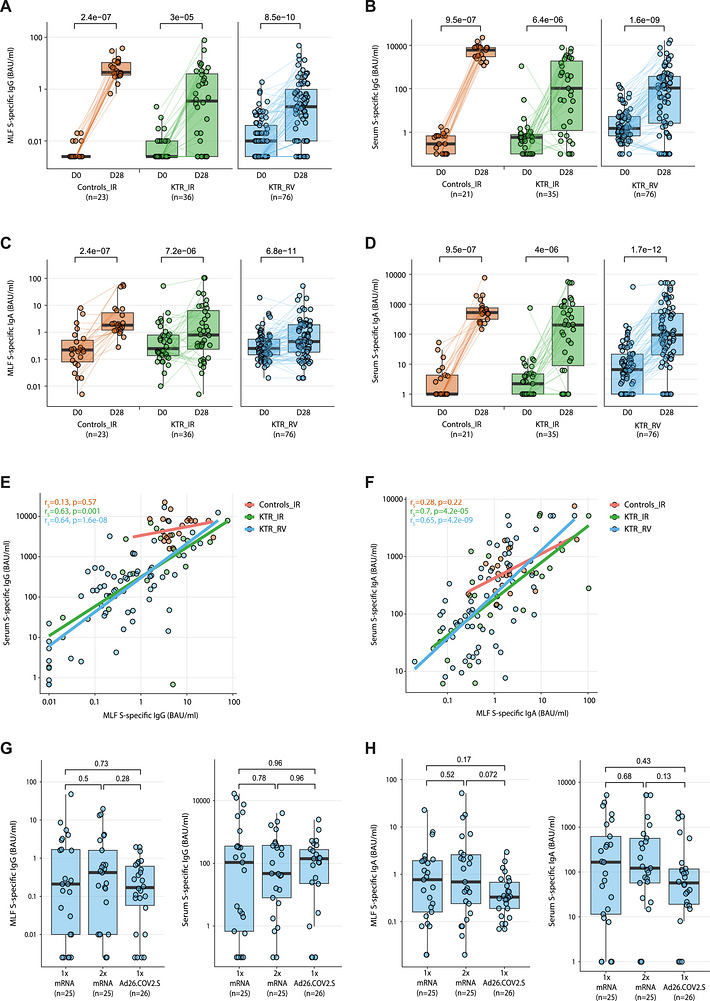
Antibody responses to COVID‐19 vaccination in controls and KTRs. (A and B) Spike (S)‐specific IgG levels in controls and KTRs at baseline and 28 days after two and repeated vaccinations, measured in nasal MLF and serum, respectively. Boxes represent the 25th and 75th percentiles, horizontal lines represent medians, and dots represent individual participant values. Differences between timepoints were assessed using the Wilcoxon signed‐rank test. (C and D) S‐specific IgA levels in controls and KTRs at baseline and 28 days after vaccination, measured in nasal MLF and serum, respectively. (E and F) Correlation between nasal and serum S‐specific IgG (E) or IgA (F) levels at D28. Participants with one or both samples below the lower limit of detection were excluded from the analysis (IgG: *N* = 107 excluded; IgA: *N* = 69 excluded). Spearman correlation coefficients (*r*_S_) and corresponding *p*‐values are shown. (G) S‐specific IgG levels in KTR‐RV subgroups at 28 days after receiving a single dose of mRNA, a double dose of mRNA, or a heterologous dose of Ad26.COV2.S. (H) S‐specific IgA levels in KTR‐RV subgroups at 28 days after receiving a single dose of mRNA, a double dose of mRNA, or a heterologous dose of Ad26.COV2.S. Controls‐IR are shown in red. 1x Ad26.COV2.S, KTR‐RV receiving a single dose of Ad26.COV2‐S; 1x mRNA, KTR‐RV receiving a single dose of mRNA‐1273; 2x mRNA, KTR‐RV receiving a double dose of mRNA‐1273; BAU, binding arbitrary units; KTR‐IR, kidney transplant recipients from the RECOVAC‐IR study (blue); KTR‐RV, kidney transplant recipients from the RECOVAC‐RV study (green); MLF, mucosal lining fluid; S, spike.

Nasal and serum samples were collected from all participants at baseline and 28 days after the second vaccination. In all groups, GMC of S‐specific IgG in both nasal and serum samples increased significantly from baseline to D28 after vaccination (Figure 1A,B). However, mucosal S‐specific IgG GMCs were significantly lower in the KTR‐IR group compared with controls who had also received two doses of mRNA‐1273 (0.18 vs. 5.71 BAU/mL at D28, *p* < 0.001). Serum S‐specific IgG responses followed a similar pattern, with significantly lower GMCs in KTR‐IR participants compared with controls at D28 (52.19 vs. 4849.40 BAU/mL, *p* < 0.0001).

For IgA, postvaccination increases in both nasal and serum S‐specific IgA GMC were observed across all study groups (Figure 1C,D). Mucosal IgA GMC tended to be lower in KTR‐IR than in controls at D28 (0.95 vs. 2.74 BAU/mL, *p* = 0.053). A significant difference was observed in serum S‐specific IgA GMC between KTR‐IR and controls (85.90 vs. 597.76 BAU/mL, *p* = 0.014).

Nasal mucosal and serum samples were also collected from KTRs before and 28 days after repeated vaccination. Several participants showed a higher S‐specific IgG or S‐specific IgA concentration at baseline of the RECOVAC‐RV study compared with baseline of the RECOVAC‐IR study, with an interval of 7–8 months and two vaccinations in between these timepoints. Similar to the RECOVAC‐IR cohort, GMCs of S‐specific IgG (Figure 1A,B) and IgA (Figure 1C,D) increased in both nasal and serum samples following repeated vaccination. GMCs in the KTR‐RV group were in a similar range to those observed in KTR‐IR participants, despite KTR‐RV participants having been classified as seronegative after two primary vaccine doses.

Next, we analyzed the relationship between nasal and serum S‐specific antibody levels. Overall, nasal and serum antibodies showed moderate‐to‐strong correlations (Figure 1E,F). Correlations between nasal and serum antibodies were statistically significant in both KTR‐IR and KTR‐RV participants (*r*_S_ = 0.63 and 0.64 for IgG and *r*_S_ = 0.70 and 0.65 for IgA, respectively). In contrast, correlations between nasal and serum antibodies in the control group were weak for both IgG and IgA (*r*_S_ = 0.13 and 0.28, respectively).

### 3.3 KTRs Generate Mucosal Neutralizing Antibodies

**FIGURE 2:**
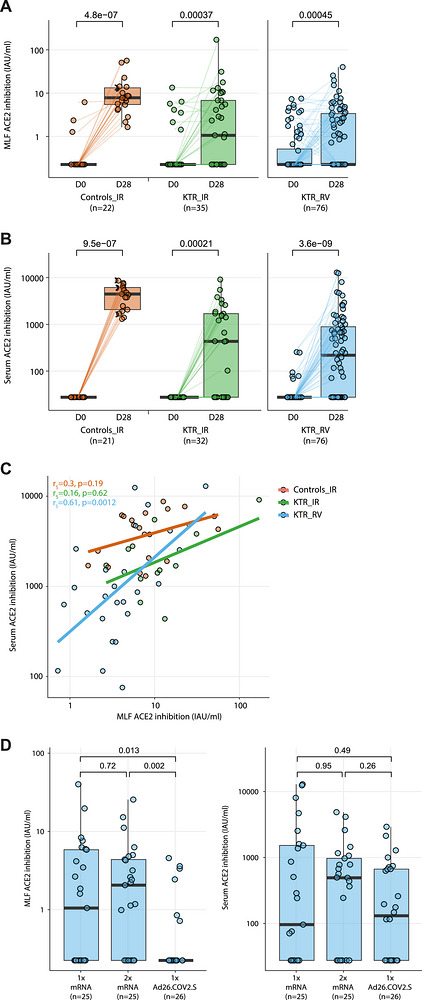
ACE2 binding inhibition responses to COVID‐19 vaccination in controls and KTRs. (A and B) ACE2 binding‐inhibiting levels in controls and KTRs at baseline and 28 days after COVID‐19 vaccination, measured in nasal MLF and serum, respectively. Boxes represent the 25th and 75th percentiles, horizontal lines represent medians, and dots represent individual participant values. Differences between timepoints were assessed using the Wilcoxon signed‐rank test. (C) Correlation between nasal and serum ACE2 binding inhibition levels at D28. Participants with one or both samples below the lower level of detection were excluded from the analysis (*N* = 203 excluded). Spearman correlation coefficients (*r*_S_) and corresponding *p*‐values are shown. (D) ACE2 binding‐inhibiting levels in KTR‐RV subgroups at 28 days after receiving a single dose of mRNA, a double dose of mRNA, or a heterologous dose of Ad26.COV2.S. IAU, inhibiting arbitrary units.

In all study groups, nasal and serum ACE2 binding inhibition levels increased significantly from baseline to D28 after vaccination (Figure 2A,B). At D28, KTR‐IR participants showed significantly lower GMCs of nasal ACE2 binding inhibition at D28 compared with controls (1.38 vs. 8.33 IAU/mL, *p* < 0.001). A similar pattern was seen in serum, with significantly lower postvaccination GMCs in KTR‐IR participants compared with controls (253.04 vs. 3860.21 IAU/mL, *p* < 0.0001).

Following repeated vaccination, KTR‐RV participants showed an increase in nasal and serum ACE2 binding inhibition, with GMCs in a range comparable to those observed in KTR‐IR participants (Figure 2A,B).

Analysis of the relationship between nasal and serum ACE2 binding inhibition revealed weak‐to‐moderate positive correlations between the two compartments (Figure 2C). Correlation coefficients were *r*_S_ = 0.30 for controls, *r*_S_ = 0.16 for KTR‐IR, and *r*_S_ = 0.61 for KTR‐RV.

### 3.4 ACE2 Binding Inhibition Correlates Strongly With IgG Responses

**FIGURE 3:**
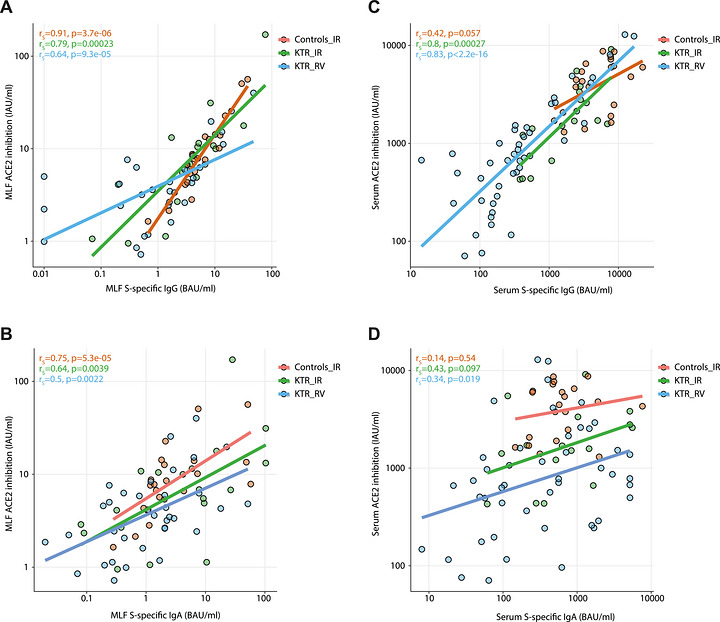
Correlation between antibody levels and ACE2 binding inhibition levels. (A, B) Correlation between nasal S‐specific IgG (A) or S‐specific IgA (B) levels and ACE2 binding inhibiting at D28. Participants with one or both samples below the lower level of detection were excluded from the analysis (*N* = 179 and 160 are excluded, respectively). (C and D) Correlation between serum S‐specific IgG (C) or IgA (D) levels and serum ACE2 binding inhibition at D28. Participants with one or both samples below the lower level of detection were excluded from the analysis (*N* = 170 and 171 are excluded, respectively). Spearman correlation coefficients (*r*_S_) and corresponding *p*‐values are shown.

To investigate the relationship between binding antibodies and ACE2 binding inhibition, we performed correlation analyses across all study groups and sample types. In nasal samples, strong and statistically significant correlations were observed between S‐specific IgG levels and ACE2 binding inhibition across all groups (*r*_S_ = 0.91, 0.79, and 0.64 for controls, KTR‐IR, and KTR‐RV, respectively) (Figure 3A). Correlations between nasal S‐specific IgA and nasal ACE2 binding inhibition were also significant, but of slight lower magnitude (*r*_S_ = 0.75, 0.64, and 0.50, respectively) (Figure 3B).

In serum, strong correlations between S‐specific IgG levels and serum ACE2 binding inhibition were observed in the KTR‐IR and KTR‐RV groups (*r*_S_ = 0.80 and 0.83, respectively) (Figure 3C). In contrast, the correlation in the control group was lower (*r*_S_ = 0.42), again likely reflecting a narrow range of uniformly high serum S‐specific IgG concentrations and ACE2 binding inhibition in these participants. Weak‐to‐moderate correlations were observed between serum S‐specific IgA and serum ACE2 binding inhibition in controls, KTR‐IR, and KTR‐RV participants (*r*_S_ = 0.14, 0.43, and 0.34, respectively) (Figure 3D).

### 3.5 Similar Responses Across Repeated Vaccination Subgroups

Previously, we observed no differences in serum S‐specific IgG at D28 after a third or fourth vaccination in KTRs who remained seronegative after two vaccinations, irrespective of whether they received a single dose of mRNA‐1273, a double dose of mRNA‐1273, or a heterologous vaccination with Ad26.COV2‐S [10].

As a sensitivity analysis, we compared nasal and serum samples collected at D28 for S‐specific IgG (Figure 1G), S‐specific IgA (Figure 1H), and ACE2 binding inhibition (Figure 2D) across these different repeated vaccination strategies. In line with our previous observation, no significant differences were detected between the subgroups for most parameters. The only exception was a significantly lower level of nasal ACE2 binding inhibition in participants who received Ad26.COV‐2S compared with those who received either a single or double dose of mRNA‐1273.

As these findings could affect correlation analyses within the KTR‐RV cohort, we performed additional subgroup analyses stratified by repeated vaccination strategy. Specifically, we examined correlations between nasal and serum antibody responses (Figure S2), as well as correlations between antibody levels and ACE2 binding inhibition (Figure S3), within each subgroup. Significant correlations were observed for all subgroups between S‐specific IgG and IgA levels in both serum and nasal samples. In contrast, correlations involving ACE2 binding inhibition were not consistently observed across subgroups. Subgroup‐specific correlation analyses involving ACE2 binding inhibition were limited by the number of samples within the quantifiable range of the assay, particularly in the Ad26.COV2‐S subgroup.

### 3.6 IgA in Nasal MLF Is Predominantly Polymeric

To assess whether IgA in nasal MLF and serum differs in its polymeric composition, Luminex beads coupled to anti‐IgA capture antibodies were incubated with MLF or serum samples and subsequently detected using either an anti‐IgA or anti‐J‐chain secondary antibody (Figure S3A). In nasal MLF, a strong correlation was observed between total IgA and J‐chain signals (*r*_S_ = 0.94), whereas serum showed a high total IgA signal with little to no detectable J‐chain signal (*r*_S_ = 0.078).

When S‐conjugated beads were used with the same secondary antibodies, IgA and J‐chain signals again correlated strongly in nasal MLF (*r*_S_ = 0.82), consistent with the presence of J‐chain‐associated S‐specific IgA in the nasal compartment (Figure S3B). In serum, a moderate correlation between S‐specific IgA and J‐chain signals was observed (*r*_S_ = 0.61).

## 4 Discussion

Effective mucosal immunity in the upper respiratory tract is essential for ensuring durable protection at the population level by restricting the spread of SARS‐CoV‐2, and at the individual level by preventing infection and progression to severe COVID‐19. Despite this, relatively little is known about mucosal antibody responses in KTRs, a major risk group for severe COVID‐19. To address this gap, we investigated nasal mucosal antibody responses to two and repeated SARS‐CoV‐2 vaccination in KTRs and assessed their relationship with circulating antibodies.

We observed a significant rise in both nasal and serum IgG and IgA antibodies directed against the viral S‐antigen following vaccination, demonstrating that KTRs are capable of mounting short‐term humoral immune responses in both compartments. In parallel, ACE2 binding inhibition increased in both serum and nasal samples, suggesting that vaccination‐induced antibodies in KTRs are functionally capable of neutralizing viral entry. Nonetheless, GMCs of S‐specific IgG and S‐specific IgA, as well as ACE2 binding inhibition, were significantly lower in KTRs than in controls following primary vaccination. These findings align with previous reports of attenuated humoral immunity in immunosuppressed populations.

Nasal antibody levels correlated strongly with serum antibody levels, consistent with previous studies in healthy individuals [21, 25, 26], and indicating that serum antibodies effectively reach the nasal mucosa in this high‐risk group. In addition, we demonstrated that nasal ACE2 binding inhibition strongly correlated nasal S‐specific IgG levels, supporting the concept that mucosal neutralizing capacity after systemic vaccination is predominantly driven by IgG.

Several studies have reported increases in nasal S‐specific IgG after primary COVID‐19 vaccination [21, 27], reflecting concomitant increases in serum IgG. It is generally thought that most mucosal IgG induced by systemic vaccination originates from the circulation and crosses the mucosal barrier via the neonatal Fc receptor (FcRN) or through passive exudation [20, 21, 28]. While relatively few studies have assessed IgA response after vaccination, those that did reported modest increases in serum IgA [29, 30]. Consistent with these findings, we observed small yet significant increases in both serum and nasal S‐specific IgA. Previous work showed that mucosal IgA responses after natural infection are substantially higher than after vaccination [22], suggesting that intramuscular vaccination is less effective at inducing local IgA responses.

Measurable S‐specific IgA was detected in a substantial fraction of participants prior to primary vaccination, despite confirmed SARS‐CoV‐2 naivety. This effect was more pronounced in nasal samples than in serum and may reflect the presence of secretory IgA, cross‐reactive antibodies, or nonspecific binding related to the secretory component or other proteins present in nasal secretions. In addition, the use of undiluted or minimally diluted MLF samples may contribute to this observation.

It has previously been shown that systemic vaccination induces nasal neutralizing antibodies in the general population [20, 28, 31]. In our study, all control participants developed detectable mucosal ACE2 inhibiting antibodies after primary vaccination. In contrast, although both KTR cohorts showed significant increases in mucosal ACE2 binding inhibition, levels remained substantially lower than in controls, with neutralizing activity remaining below the assay detection limit in a subset of KTRs. Notably, mucosal ACE2 binding inhibition after repeated vaccination was lower in participants receiving heterologous Ad26.COV2‐S compared with homologous mRNA‐1273 booster regimens. This observation is consistent with prior reports showing lower serum neutralizing antibody responses following adenoviral vector‐based vaccination, both as primary and booster regimens [32, 33, 34]. Nonetheless, adenoviral vectors have been used as mucosal vaccine platforms with varying success so far in nonhuman primates [35]. Our data indicate that the use of intramuscular injections with adenoviral vectors does not predict the potential effective use of mucosal adenoviral vector vaccine platforms in the future. However, subgroup‐specific analyses were limited by the number of samples within the quantifiable range of the ACE2 inhibition assay, particularly in the Ad26.COV2‐S subgroup, which restricts the strength of conclusions that can be drawn regarding booster‐specific differences in mucosal neutralization. Furthermore, the ACE2 binding inhibition assay does not distinguish between antibody isotypes, and both S‐specific IgG and IgA show an increase 28 days after vaccination. Previously, we showed that the contribution of mucosal antibodies to ACE2 inhibition differs by type of primary exposure, with neutralization after vaccination being predominantly driven by mucosal IgG [22].

To assess mucosal immunity, we employed nasosorption to collect MLF, a minimally invasive sampling method compared with nasopharyngeal aspiration, swabs, or venipuncture [17, 36, 37, 38]. This approach facilitates repeated sampling and may be particularly valuable in vulnerable populations or remote settings [17]. However, the nasal cavity does not fully represent immune responses throughout the respiratory tract, and extrapolation to lower airway immunity should be done cautiously [39]. Furthermore, it was previously unclear whether mucosal IgA after intramuscular vaccination is derived from serum or locally produced, particularly in KTR. In exploratory experiments, the strong correlation between total IgA and J‐chain signal in nasal MLF, combined with the absence of J‐chain signal in serum, suggests that nasal IgA is predominantly polymeric, whereas circulating IgA is largely monomeric. Importantly, J‐chain‐associated S‐specific IgA was also detected in nasal MLF. As polymeric J‐chain‐containing IgA is not the predominant form in serum, these findings suggest that S‐specific IgA in the nasal compartment may at least in part be locally produced, though formal confirmation requires detection of the secretory component. Of note, a contribution of S‐specific IgM to the J‐chain signal in serum cannot be excluded.

Several limitations should be considered. First, we assessed neutralizing activity using an ACE2 binding inhibition assay rather than live‐virus neutralization assays such as the plaque reduction neutralization test (PRNT), which remains the gold standard [40]. Nonetheless, we have previously demonstrated a strong correlation (*R* > 0.9) between serum ACE2 binding inhibition assay and PRNT results [22]. Furthermore, MLF ACE2 binding inhibition at near‐threshold values should be interpreted with caution. Second, other antibody effector functions, including antibody‐dependent cellular cytotoxicity (ADCP) and phagocytosis (ADCP), were not assessed and may also contribute to protection [32, 41, 42]. Technical constraints, including low antibody concentrations and limited sample volumes in MLF, currently limit the feasibility of such assays in mucosal samples. Third, all vaccines administered and immunological assays were based on the ancestral Wuhan‐Hu‐1 S protein. Reduced vaccine effectiveness against Omicron sublineages has been reported due to immune imprinting with recall against the Wuhan‐Hu‐1 strain [22, 43], and future studies evaluating current protection levels should incorporate variant‐adapted S‐antigens.

Mucosal cellular immune responses may become increasingly important as antibody levels wane. While local CD4+ and CD8+ T cell responses in the upper airways have been associated with reduced disease severity [27], evidence regarding the induction of mucosal cellular responses after mRNA vaccination remains mixed [20, 44, 45]. Solid organ transplant recipients, in particular, are known to have impaired cellular immune response and remain at an elevated risk for breakthrough infections [46, 47]. As mucosal T‐ and B‐cellular responses were not assessed in the RECOVAC studies, this important aspect warrants further investigation.

Finally, we did not systematically monitor SARS‐CoV‐2 infections, viral loads, longer‐term kinetics, or clinical outcomes, precluding direct linkage of mucosal ACE2 binding inhibition levels to protection from infection or disease severity in KTRs.

In conclusion, our data demonstrate that KTRs can mount functional, ACE2‐inhibiting nasal mucosal antibodies shortly after intramuscular mRNA vaccination, although these responses are quantitatively lower than those observed in controls. The strong correlation between mucosal and systemic antibody responses supports the use of nasal antibody measurements as a minimally invasive supplement to serum‐based analyses. Together, these data provide a rationale for evaluating mucosal vaccination strategies as a means to further improve local immunity in immunocompromised populations.

## Author Contributions

R.T.G., J.‐S.F.S., and L.B.H. designed the study protocol. A.L.M., F.J.B., M.M.L.K., C.C.B., D.v.B., R.v.B., G.d.H., R.G.v.d.M., and D.A.D. contributed to the protocol design. V.J.C.H.K., R.P., M.C.B., R.G.v.d.M., L.B.H., and D.A.D. participated in obtaining the samples. V.J.C.H.K., C.E.v.d.G.‐d.J., and F.v.O. contributed to the lab processing. V.J.C.H.K., R.G.v.d.M., L.B.H., and D.A.D. participated in the data analysis. V.J.C.H.K. wrote the original draft. V.J.C.H.K., R.G.v.d.M., L.B.H., and D.A.D. participated in reviewing and editing the manuscript. A.L.M., M.M.L.K., J.‐S.F.S., R.v.B., G.d.H., and R.T.G. contributed to the intellectual content.

## Conflicts of Interest

The authors declare no conflicts of interest.

## Supporting information




**Supporting File 1**: tid70230‐sup‐0001‐figuresS1‐S4.pdf.

**Supporting File 2**: tid70230‐sup‐0002‐tableS1.docx.

**Supporting File 3**: tid70230‐sup‐0003‐VisualAbstract.pdf.

## Data Availability

The data and R‐code that support the findings of this study are available from the corresponding author upon reasonable request. The raw data are not publicly available due to data and patients' privacy laws.

## References

[1] E. J. Williamson , A. J. Walker , K. Bhaskaran , et al., “OpenSAFELY: Factors Associated With COVID‐19 Death in 17 Million Patients,” Nature 584, no. 7821 (2021): 430–436. 10.1038/s41586-020-2521-4 PMC7611074 32640463

[2] J.‐S. F. Sanders , F. J. Bemelman , A. L. Messchendorp , et al., “The RECOVAC Immune‐Response Study: The Immunogenicity, Tolerability, and Safety of COVID‐19 Vaccination in Patients With Chronic Kidney Disease, on Dialysis, or Living With a Kidney Transplant,” Transplantation 106, no. 4 (2022): 821–834. 34753894 10.1097/TP.0000000000003983 PMC8942603

[3] H. Rincon‐Arevalo , M. Choi , A.‐L. Stefanski , et al., “Impaired Humoral Immunity to SARS‐CoV‐2 BNT162b2 Vaccine in Kidney Transplant Recipients and Dialysis Patients,” Science Immunology 6, no. 60 (2021): eabj1031. 34131023 10.1126/sciimmunol.abj1031

[4] D. Yelin , B. Rozen‐Zvi , D. Yahav , et al., “SARS‐CoV‐2 Antibody Dynamics Among Kidney Transplant Recipients 3 Months After BNT162b2 Vaccination: a Prospective Cohort Study,” Clinical Kidney Journal 15, no. 5 (2022): 992–998. 35498878 10.1093/ckj/sfac031 PMC8903319

[5] B. Rozen‐Zvi , D. Yahav , T. Agur , et al., “Antibody Response to SARS‐CoV‐2 mRNA Vaccine Among Kidney Transplant Recipients: a Prospective Cohort Study,” Clinical Microbiology and Infection 27, no. 8 (2021): 1173.e1–1173.e4. 10.1016/j.cmi.2021.04.028 PMC8091803 33957273

[6] C. Watschinger , G. Stampfel , A. Zollner , et al., “B and T Cell Responses to SARS‐CoV‐2 Vaccination in Kidney and Liver Transplant Recipients With and Without Previous COVID‐19,” Viruses 16, no. 1 (2023): 1. 38275936 10.3390/v16010001 PMC10820906

[7] Z. A. Fatly , M. G. H. Betjes , W. A. Dik , R. A. M. Fouchier , M. E. J. Reinders , and A. E. de Weerd , “Mycophenolate Mofetil Hampers Antibody Responses to a Broad Range of Vaccinations in Kidney Transplant Recipients: Results From a Randomized Controlled Study,” Journal of Infection 88, no. 3 (2024): 106133. 38432583 10.1016/j.jinf.2024.106133

[8] J. Stumpf , T. Siepmann , J. Schwöbel , et al., “MMF/MPA Is the Main Mediator of a Delayed Humoral Response With Reduced Antibody Decline in Kidney Transplant Recipients After SARS‐CoV‐2 mRNA Vaccination,” Frontiers in Medicine 9 (2022): 928542. 35872777 10.3389/fmed.2022.928542 PMC9300891

[9] V. López , C. Polo , R. Schuldt , et al., “Humoral Response After SARS‐CoV‐2 Vaccination in Kidney Transplant Recipients: Role of Immunosuppression Therapy,” Transplantation Proceedings 54, no. 9 (2022): 2454–2456. 36273957 10.1016/j.transproceed.2022.09.019 PMC9527195

[10] M. M. L. Kho , A. L. Messchendorp , S. C. Frölke , et al., “Alternative Strategies to Increase the Immunogenicity of COVID‐19 Vaccines in Kidney Transplant Recipients Not Responding to Two or Three Doses of an mRNA Vaccine (RECOVAC): a Randomised Clinical Trial,” Lancet Infectious Diseases 23, no. 3 (2023): 307–319. 36354032 10.1016/S1473-3099(22)00650-8 PMC9760034

[11] S. R. K. Malahe , Y. den Hartog , W. J. R. Rietdijk , et al., “Repeated COVID‐19 Vaccination Drives Memory T‐ and B‐Cell Responses in Kidney Transplant Recipients: Results From a Multicenter Randomized Controlled Trial,” Transplantation 108, no. 12 (2024): 2420–2433. 38902860 10.1097/TP.0000000000005119 PMC11581438

[12] D. Hauser , L. Urda , C. Lang , et al., “Benefits of Repeated SARS‐CoV‐2 Vaccination and Virus‐Induced Cross‐Neutralization Potential in Immunocompromised Transplant Patients and Healthy Individuals,” Open Forum Infectious Diseases 11, no. 10 (2024): ofae527. 39371367 10.1093/ofid/ofae527 PMC11450466

[13] W. A. Werbel , A. H. Karaba , T. P.‐Y. Chiang , et al., “Persistent SARS‐CoV‐2–Specific Immune Defects in Kidney Transplant Recipients Following Third mRNA Vaccine Dose,” American Journal of Transplantation 23, no. 6 (2023): 744–758. 36966905 10.1016/j.ajt.2023.03.014 PMC10037915

[14] O. Gallo , L. G. Locatello , A. Mazzoni , L. Novelli , and F. Annunziato , “The Central Role of the Nasal Microenvironment in the Transmission, Modulation, and Clinical Progression of SARS‐CoV‐2 Infection,” Mucosal Immunology 14, no. 2 (2021): 305–316. 33244161 10.1038/s41385-020-00359-2 PMC7690066

[15] M. W. Russell , Z. Moldoveanu , P. L. Ogra , and J. Mestecky , “Mucosal Immunity in COVID‐19: a Neglected but Critical Aspect of SARS‐CoV‐2 Infection,” Frontiers in Immunology 11 (2020): 611337. 33329607 10.3389/fimmu.2020.611337 PMC7733922

[16] M. K. Verheul , J. Kaczorowska , M. I. Hofstee , et al., “Protective Mucosal SARS‐CoV‐2 Antibodies in the Majority of the General Population in the Netherlands,” Mucosal Immunology 17, no. 4 (2024): 554–564. 38553008 10.1016/j.mucimm.2024.03.008

[17] J. Fröberg , J. Gillard , R. Philipsen , et al., “SARS‐CoV‐2 Mucosal Antibody Development and Persistence and Their Relation to Viral Load and COVID‐19 Symptoms,” Nature Communications 12, no. 1 (2021): 5621. 10.1038/s41467-021-25949-x PMC8460778 34556667

[18] K. Sano , D. Bhavsar , G. Singh , et al., “SARS‐CoV‐2 Vaccination Induces Mucosal Antibody Responses in Previously Infected Individuals,” Nature Communications 13, no. 1 (2022): 5135. 10.1038/s41467-022-32389-8 PMC9435409 36050304

[19] M. Guerrieri , B. Francavilla , D. Fiorelli , et al., “Nasal and Salivary Mucosal Humoral Immune Response Elicited by mRNA BNT162b2 COVID‐19 Vaccine Compared to SARS‐CoV‐2 Natural Infection,” Vaccines 9, no. 12 (2021): 1499. 34960244 10.3390/vaccines9121499 PMC8708818

[20] J. Tang , C. Zeng , T. M. Cox , et al., “Respiratory Mucosal Immunity Against SARS‐CoV‐2 After mRNA Vaccination,” Science Immunology 7, no. 76 (2022): eadd4853. 35857583 10.1126/sciimmunol.add4853 PMC9348751

[21] K. T. Cao , C. Cobos‐Uribe , N. Knight , et al., “SARS‐CoV‐2 mRNA Vaccination Induces an Intranasal Mucosal Response Characterized by Neutralizing Antibodies,” Journal of Allergy and Clinical Immunology: Global 2, no. 4 (2023): 100129. 37781659 10.1016/j.jacig.2023.100129 PMC10290737

[22] J. Fröberg , V. J. C. H. Koomen , C. E. van der Gaast‐de Jongh , et al., “Primary Exposure to SARS‐CoV‐2 via Infection or Vaccination Determines Mucosal Antibody‐Dependent ACE2 Binding Inhibition,” Journal of Infectious Diseases 229, no. 1 (2024): 137–146. 37675756 10.1093/infdis/jiad385 PMC10786246

[23] M. M. L. Kho , M. E. J. Reinders , C. C. Baan , et al., “The RECOVAC IR Study: The Immune Response and Safety of the mRNA‐1273 COVID‐19 Vaccine in Patients With Chronic Kidney Disease, on Dialysis or Living With a Kidney Transplant,” Nephrology Dialysis Transplantation 36, no. 9 (2021): 1761–1764. 10.1093/ndt/gfab186 PMC8241423 34450647

[24] WHO , "First WHO International Standard for Anti‐SARS‐CoV‐2 Immunoglobulin (Human)," 2020, https://www.who.int/teams/health‐product‐policy‐and‐standards/standards‐and‐specifications/norms‐and‐standards/vaccine‐standardization/coronavirus‐disease‐(covid‐19). 10.1016/S0140-6736(21)00527-4 PMC7987302 33770519

[25] O. Puhach , M. Bellon , K. Adea , et al., “SARS‐CoV‐2 Convalescence and Hybrid Immunity Elicits Mucosal Immune Responses,” EBioMedicine 98 (2023): 104893. 38035462 10.1016/j.ebiom.2023.104893 PMC10755109

[26] O. Bladh , K. Aguilera , U. Marking , et al., “Comparison of SARS‐CoV‐2 Spike‐Specific IgA and IgG in Nasal Secretions, Saliva and Serum,” Frontiers in Immunology 15 (2024): 1346749. 38558811 10.3389/fimmu.2024.1346749 PMC10978617

[27] E. Mitsi , M. O. Diniz , J. Reiné , et al., “Respiratory Mucosal Immune Memory to SARS‐CoV‐2 After Infection and Vaccination,” Nature Communications 14, no. 1 (2023): 6815. 10.1038/s41467-023-42433-w PMC10603102 37884506

[28] J. Declercq , S. Gerlo , S. Van Nevel , et al., “Repeated COVID‐19 mRNA‐Based Vaccination Contributes to SARS‐CoV‐2 Neutralizing Antibody Responses in the Mucosa,” Science Translational Medicine 16, no. 770 (2024): eadn2364. 39441904 10.1126/scitranslmed.adn2364

[29] R. Fraser , A. Orta‐Resendiz , A. Mazein , and D. H. Dockrell , “Upper Respiratory Tract Mucosal Immunity for SARS‐CoV‐2 Vaccines,” Trends in Molecular Medicine 29, no. 4 (2023): 255–267. 36764906 10.1016/j.molmed.2023.01.003 PMC9868365

[30] O. Bladh , U. Marking , S. Havervall , et al., “Mucosal Immune Responses Following a Fourth SARS‐CoV‐2 Vaccine Dose,” The Lancet Microbe 4, no. 7 (2023): e488. 37086736 10.1016/S2666-5247(23)00102-7 PMC10115585

[31] J. Declercq , E. Tobback , S. Vanhee , et al., “COVID‐19 Vaccination With BNT162b2 and ChAdOx1 Vaccines Has the Potential to Induce Nasal Neutralizing Antibodies,” Allergy 77, no. 1 (2022): 304–307. 34543444 10.1111/all.15101 PMC8653144

[32] H. T. Zedan , M. K. Smatti , D. W. Al‐Sadeq , et al., “SARS‐CoV‐2 Infection Triggers More Potent Antibody‐Dependent Cellular Cytotoxicity (ADCC) Responses Than mRNA‐, Vector‐, and Inactivated Virus‐Based COVID‐19 Vaccines,” Journal of Medical Virology 96, no. 3 (2024): e29527. 38511514 10.1002/jmv.29527

[33] V. Naranbhai , W. F. Garcia‐Beltran , C. C. Chang , et al., “Comparative Immunogenicity and Effectiveness of mRNA‐1273, BNT162b2 and Ad26.COV2.S COVID‐19 Vaccines,” Journal of Infectious Diseases 225:(2021): 1141–1150. 10.1093/infdis/jiab593 PMC8689763 34888672

[34] M. Le Gars , J. Sadoff , V. Cárdenas , et al., “Safety, Reactogenicity, and Immunogenicity of Ad26.COV2.S as Homologous or Heterologous COVID‐19 Booster Vaccination: Results of a Randomized, Double‐Blind, Phase 2 Trial,” Vaccine 42, no. 19 (2024): 3938–3952. 38918103 10.1016/j.vaccine.2024.03.079

[35] M. Gagne , B. J. Flynn , S. F. Andrew , et al., “Mucosal Adenovirus Vaccine Boosting Elicits IgA and Durably Prevents XBB.1.16 Infection in Nonhuman Primates,” Nature Immunology 25, no. 10 (2024): 1913–1927. 39227514 10.1038/s41590-024-01951-5 PMC11436372

[36] M. A. Baumgartner , C. Li , T. M. Kuntz , et al., “Differences of the Nasal Microbiome and Mycobiome by Clinical Characteristics of COPD Patients,” Chronic Obstructive Pulmonary Diseases: Journal of the COPD Foundation 9, no. 3 (2022): 309–324. 35487694 10.15326/jcopdf.2021.0267 PMC9448003

[37] R. S. Thwaites , K. Ito , J. M. S. Chingono , et al., “Nasosorption as a Minimally Invasive Sampling Procedure: Mucosal Viral Load and Inflammation in Primary RSV Bronchiolitis,” Journal of Infectious Diseases 215, no. 8 (2017): 1240–1244. 28368490 10.1093/infdis/jix150 PMC5441107

[38] M. E. Rebuli , A. M. Speen , and P. W. Clapp , “Novel Applications for a Noninvasive Sampling Method of the Nasal Mucosa,” American Journal of Physiology‐Lung Cellular and Molecular Physiology 312, no. 2 (2017): L288–L296. 28011618 10.1152/ajplung.00476.2016 PMC5336583

[39] M. A. Greeley , “Immunopathology of the Respiratory System,” Immunopathology in Toxicology and Drug Development 29 (2017): 419–453.

[40] K. T. Liu , Y. J. Han , G. H. Wu , K. A. Huang , and P. N. Huang , “Overview of Neutralization Assays and International Standard for Detecting SARS‐CoV‐2 Neutralizing Antibody,” Viruses 14, no. 7 (2022): 1560. 35891540 10.3390/v14071560 PMC9322699

[41] K. Hagemann , K. Riecken , J. M. Jung , et al., “Natural Killer Cell‐Mediated ADCC in SARS‐CoV‐2‐Infected Individuals and Vaccine Recipients,” European Journal of Immunology 52, no. 8 (2022): 1297–1307. 35416291 10.1002/eji.202149470 PMC9087393

[42] J. Klingler , G. S. Lambert , V. Itri , et al., “Detection of Antibody Responses Against SARS‐CoV‐2 in Plasma and Saliva From Vaccinated and Infected Individuals,” Frontiers in Immunology 12 (2021): 759688. 34987505 10.3389/fimmu.2021.759688 PMC8721203

[43] N. Wu , K. Joyal‐Desmarais , P. A. B. Ribeiro , et al., “Long‐Term Effectiveness of COVID‐19 Vaccines Against Infections, Hospitalisations, and Mortality in Adults: Findings From a Rapid Living Systematic Evidence Synthesis and Meta‐Analysis up to December, 2022,” Lancet Respiratory Medicine 11, no. 5 (2023): 439–452. 36780914 10.1016/S2213-2600(23)00015-2 PMC9917454

[44] A. Ssemaganda , H. M. Nguyen , F. Nuhu , et al., “Expansion of Cytotoxic Tissue‐Resident CD8^+^ T Cells and CCR6^+^CD161^+^ CD4^+^ T Cells in the Nasal Mucosa Following mRNA COVID‐19 Vaccination,” Nature Communications 13, no. 1 (2022): 3357. 10.1038/s41467-022-30913-4 PMC9186487 35688805

[45] J. M. E. Lim , A. T. Tan , N. Le Bert , S. K. Hang , J. G. H. Low , and A. Bertoletti , “SARS‐CoV‐2 Breakthrough Infection in Vaccinees Induces Virus‐Specific Nasal‐Resident CD8+ and CD4+ T Cells of Broad Specificity,” Journal of Experimental Medicine 219, no. 10 (2022): e20220780. 35972472 10.1084/jem.20220780 PMC9386509

[46] M. V. Thygesen , C. Strandhave , J. M. Kiib , et al., “Correlates of SARS‐CoV‐2 Breakthrough Infections in Kidney Transplant Recipients Following a Third SARS‐CoV‐2 mRNA Vaccine Dose,” Vaccines 13, no. 8 (2025): 777. 40872864 10.3390/vaccines13080777 PMC12389881

[47] P. A. Grossi , P. Burra , E. Cozzi , et al., “An Update on SARS‐CoV‐2 Prevention Strategy in Solid Organ Transplant Recipients: An Expert Opinion,” Transplantation Reviews 39, no. 4 (2025): 100966. 41067176 10.1016/j.trre.2025.100966

