# Supplementary material for: Kidney Transplant Recipients Develop Nasal Mucosal Antibodies to SARS‐CoV‐2 With ACE2‐Inhibiting Activity Following mRNA Vaccination
Source: Transpl Infect Dis. 2026 May 7;28(4):e70230. doi: 10.1111/tid.70230 (PMC13450280; doi:10.1111/tid.70230)
Supplement: Supplementary file 1 [file TID-28-e70230-s002.pdf]

Kidney transplant recipients develop nasal mucosal antibodies to SARS-CoV-2 with ACE2-inhibiting activity following mRNA vaccination

@TheTxIDJournal

Koomen *et al*. ***Transplant Infectious Diseases***. 2025

Study design

Low serum Spike-specific antibody levels are found in kidney transplant recipients (KTR) after COVID-19 vaccination. Not much is known about their mucosal response

Response after two doses of mRNA-1273 in controls and KTR, and to an additional vaccination with one/two doses of mRNA-1273 or one dose of Ad26.COV2-S in KTR

Quantification of IgG and IgA binding to the Spike-antigen as well as antibody-dependent ACE2 binding inhibition in nasal fluid and serum

Results

- KTR show an increase in both nasal and serum antibody levels after two doses and repeated vaccination, but this response remains lower than in the control group
- There are detectable levels of nasal virus-neutralizing antibodies in KTR after vaccination
- Nasal virus-neutralization is moderately to highly correlated with nasal antibody levels

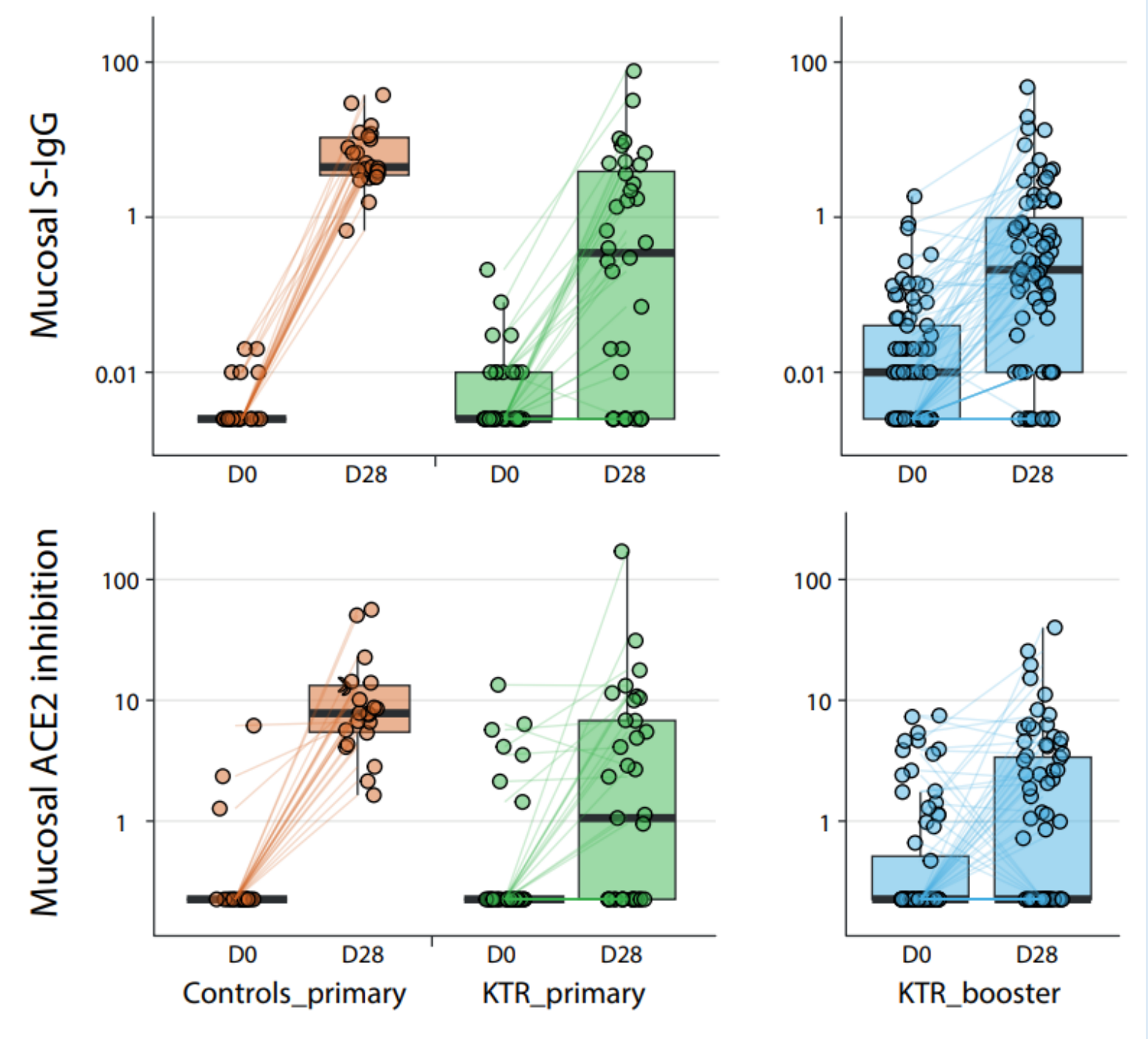

Conclusion

Even though antibody levels in kidney transplant recipients are much lower compared to the general population, this risk group is still able to generate a (functional) mucosal antibody response that might help towards protection against (re)infection

WILEY

TRANSPLANT INFECTIOUS DISEASE

The Official Journal of Transplant Infectious Disease

TX NETWORK Transplant Peer Review Network
