# Supplementary material for: Kidney Transplant Recipients Develop Nasal Mucosal Antibodies to SARS‐CoV‐2 With ACE2‐Inhibiting Activity Following mRNA Vaccination
Source: Transpl Infect Dis. 2026 May 7;28(4):e70230. doi: 10.1111/tid.70230 (PMC13450280; doi:10.1111/tid.70230)
Supplement: Supplementary file 2 [file TID-28-e70230-s003.pdf]

A

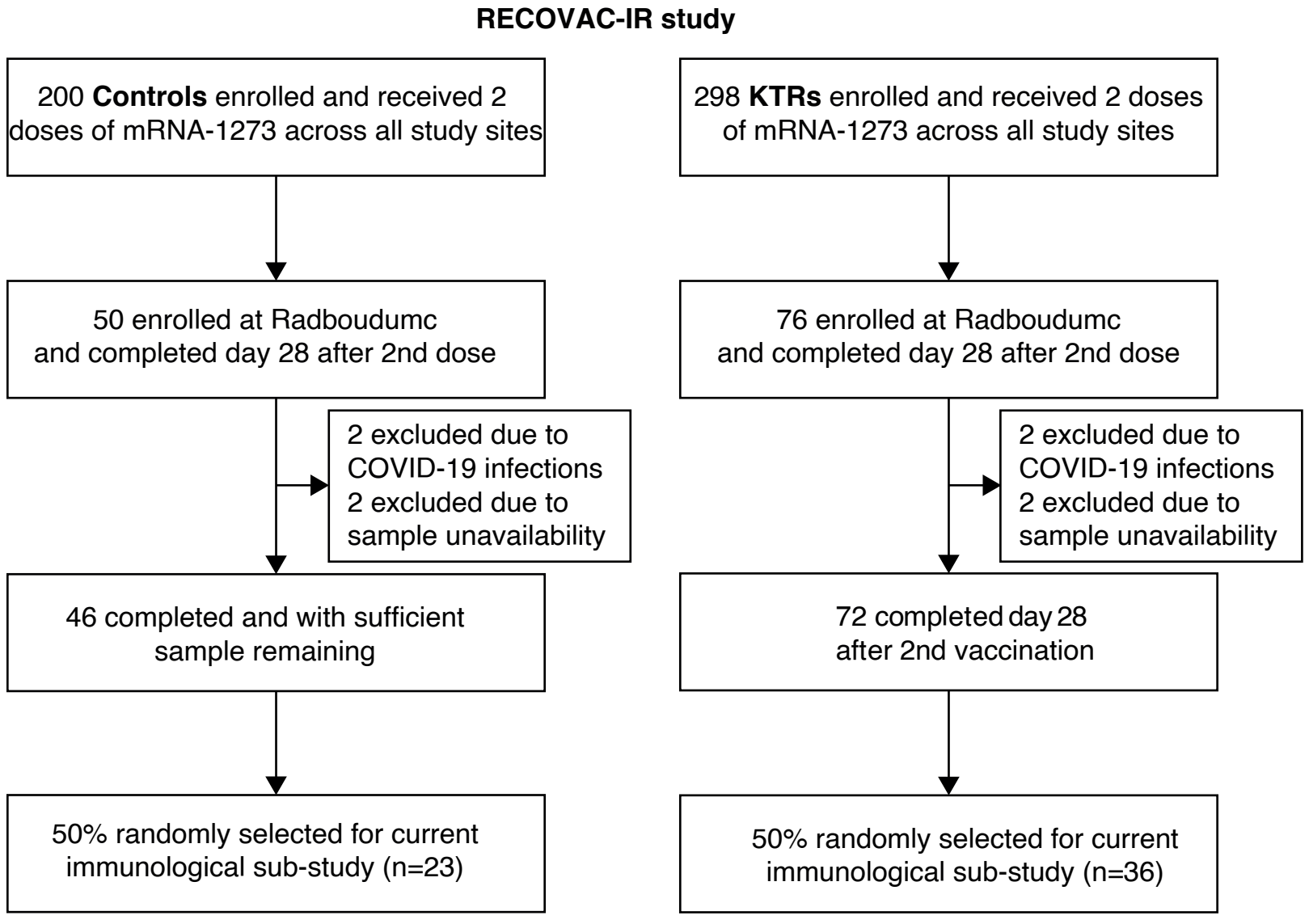

B

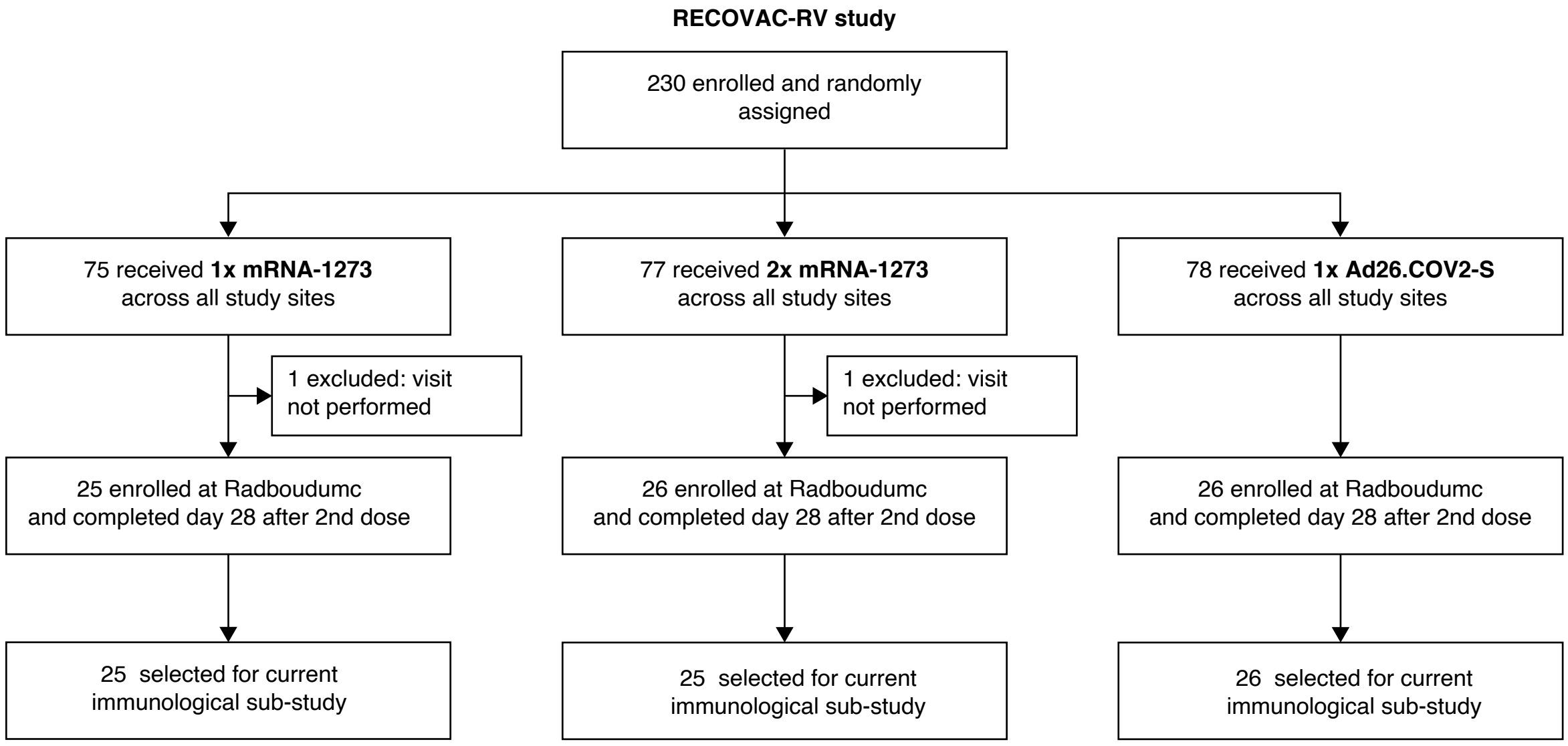

Supplementary Figure 1

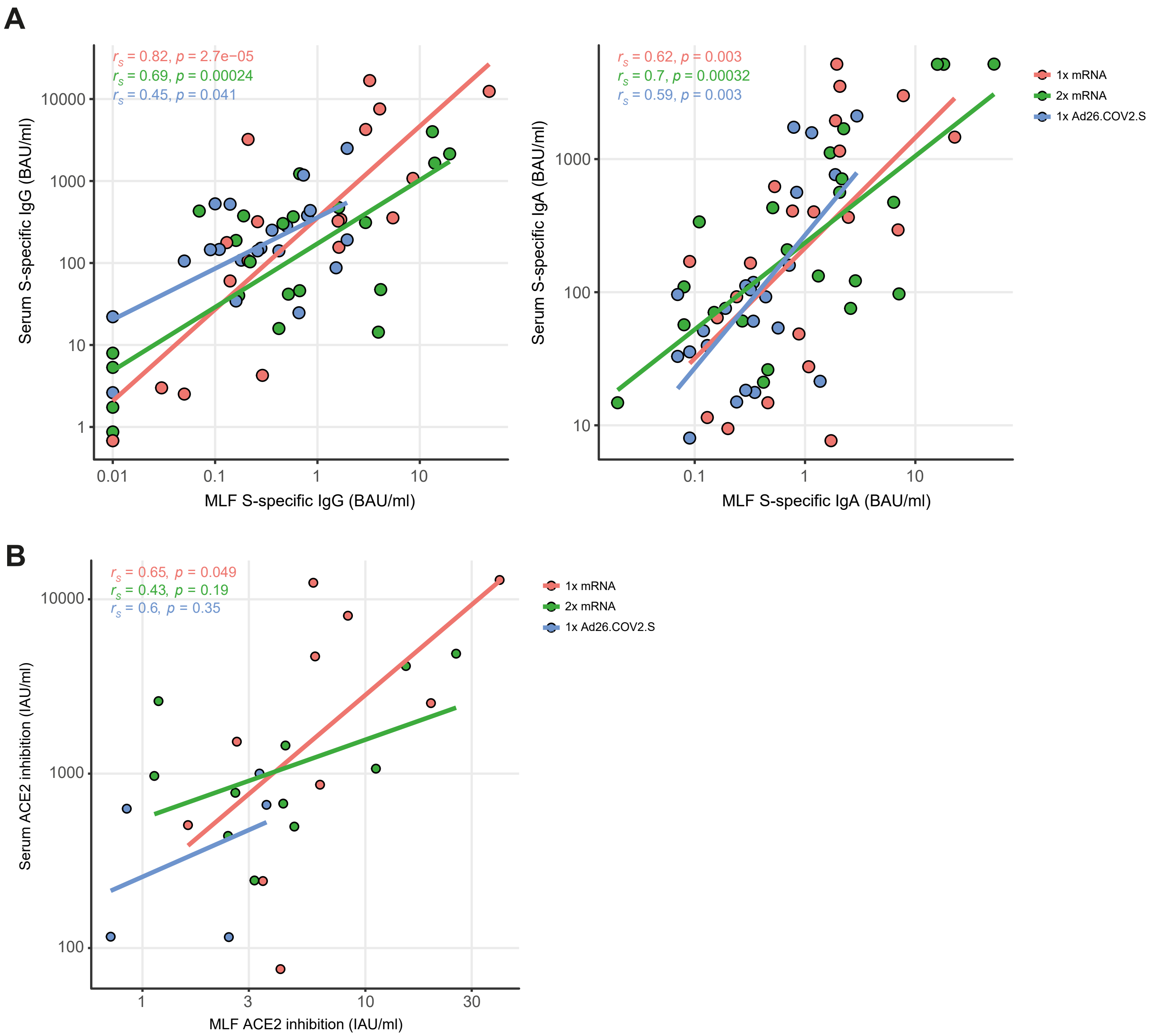

Supplementary Figure 2

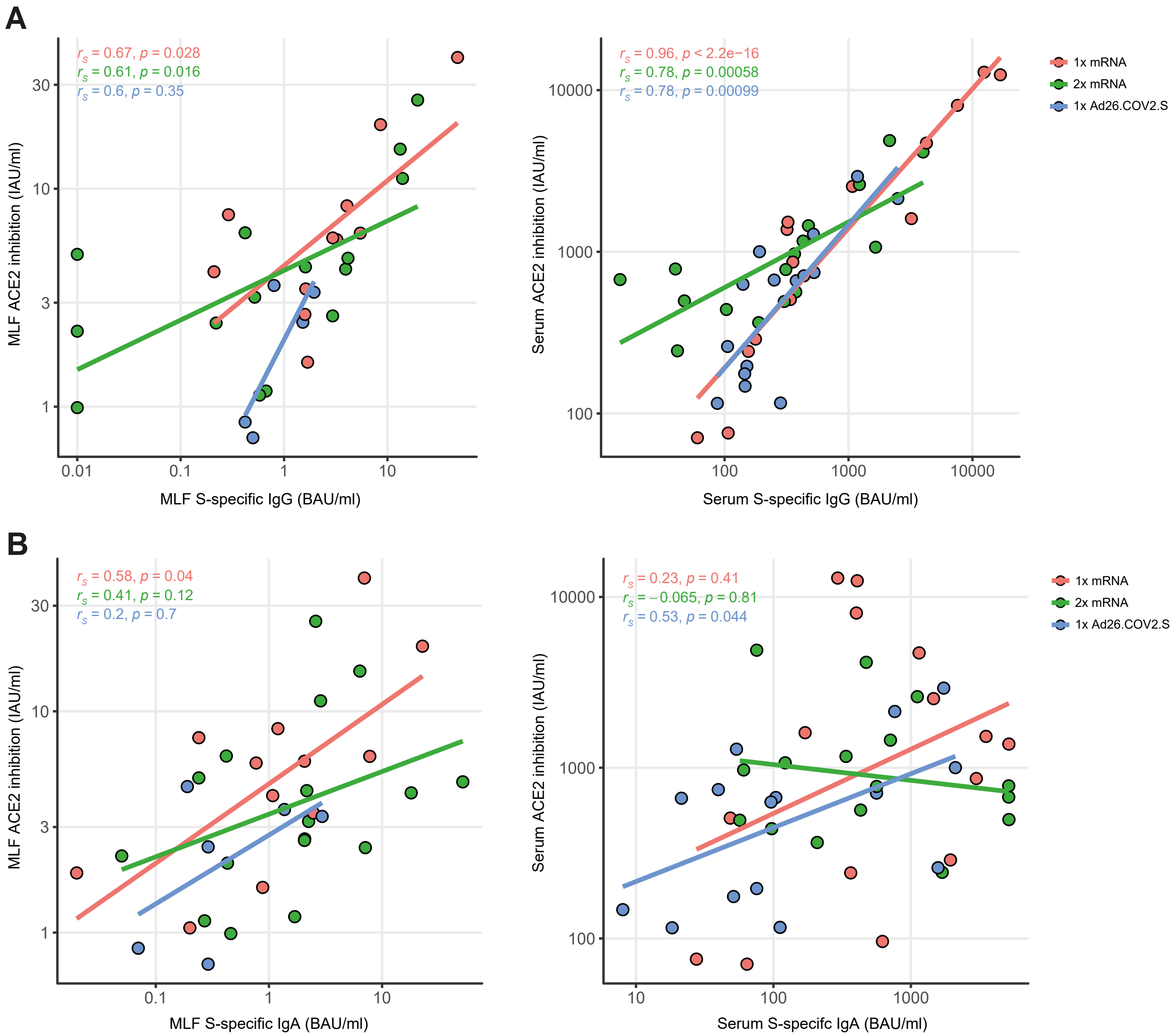

Supplementary Figure 3

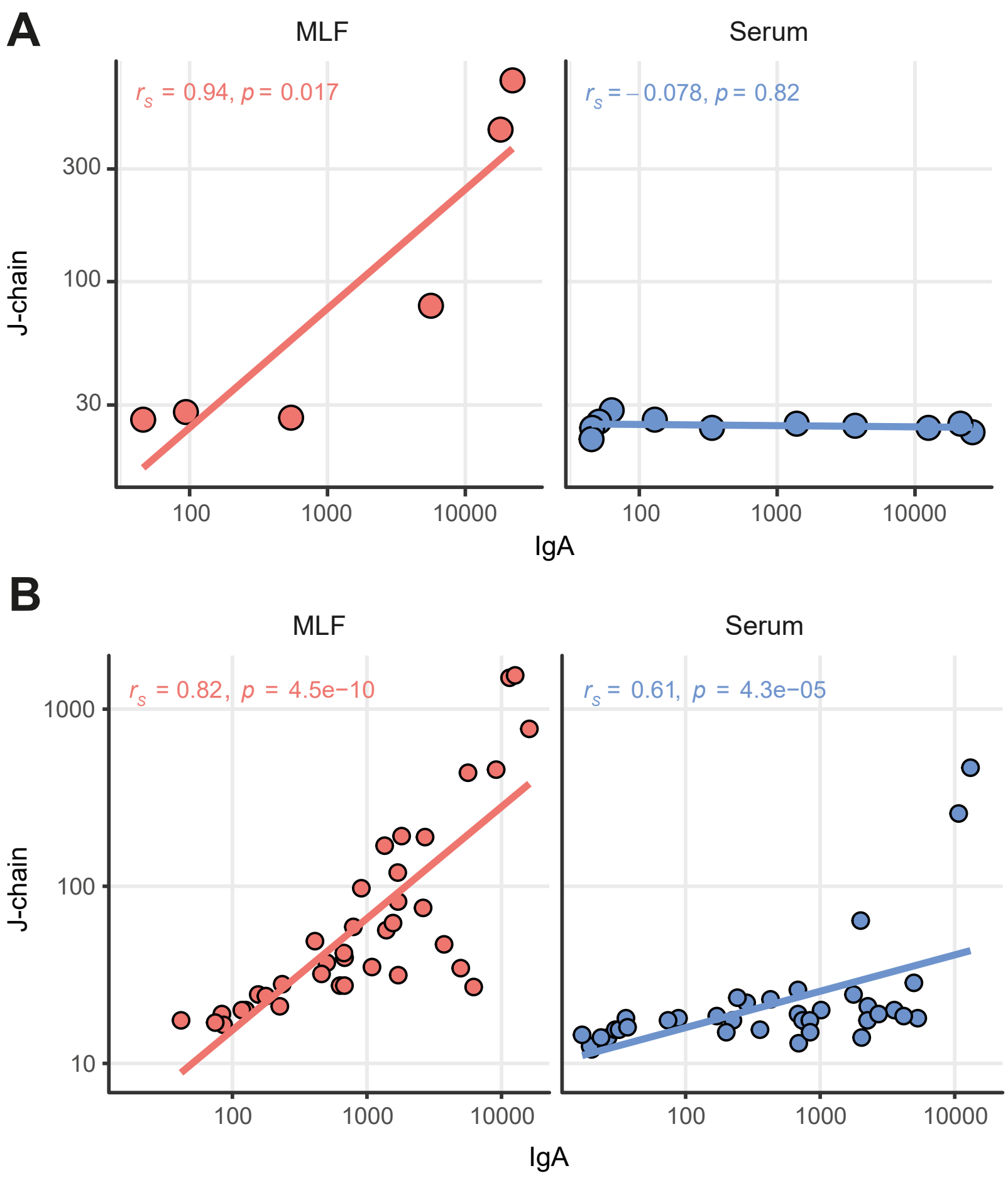

Supplementary Figure 4
