# Supplementary material for: Kidney Transplant Recipients Develop Nasal Mucosal Antibodies to SARS‐CoV‐2 With ACE2‐Inhibiting Activity Following mRNA Vaccination
Source: Transpl Infect Dis. 2026 May 7;28(4):e70230. doi: 10.1111/tid.70230 (PMC13450280; doi:10.1111/tid.70230)
Supplement: Supplementary file 3 [file TID-28-e70230-s001.docx]

|  | **1x mRNA-1273**  **(N = 25)** | **2x mRNA-1273**  **(N = 25)** | **1x Ad26.COV2.S**  **(N = 26)** |
| --- | --- | --- | --- |
| **Age** (y), median (range) | 57 (31-74) | 61 (46-77) | 60 (28-77) |
| **Female**, n (%) | 13 (52.0) | 9 (36.0) | 9 (34.6) |
| **Caucasian**, n (%) | 24 (96.0) | 24 (96.0) | 26 (100.0) |
| **BMI** (kg/m^2^), median (range) | 25.6 (17.4 – 45.6) | 25.2 (19.5 – 35.5) | 24.1 (18.7 – 32.2) |
| **Years since transplant**, median (range) | 5 (0-35) | 5 (1-24) | 6 (0-23) |
| **Immunosuppressive medication**, n (%) |  |  |  |
| Mycophenolate mofetil | 16 (64.0) | 17 (68.0) | 15 (57.7) |
| Azathioprine | 3 (12.0) | 2 (8.0) | 0 (0.0) |
| Calcineurin inhibitors | 19 (76.0) | 19 (76.0) | 21 (80.8) |
| mTOR inhibitors | 2 (8.0) | 2 (8.0) | 1 (3.8) |
| Steroids | 14 (56.0) | 16 (64.0) | 18 (69.2) |
| Three-drug regimen | 4 (16.0) | 6 (24.0) | 4 (15.4) |
| **Comorbidities**, n (%) |  |  |  |
| Hypertension | 22 (88.0) | 21 (84.0) | 24 (92.3) |
| Diabetes Mellitus | 12 (48.0) | 7 (28.0) | 7 (26.9) |
| History of coronary artery disease | 4 (16.0) | 1 (4.0) | 3 (11.5) |
| Heart failure | 0 (0.0) | 0 (0.0) | 1 (3.8) |
| Chronic lung disease | 3 (12.0) | 3 (12.0) | 4 (15.4) |
| History of malignancy | 4 (16.0) | 6 (24.0) | 6 (23.1) |
| Auto-immune disease | 1 (4.0) | 0 (0.0) | 2 (7.7) |

Supplementary Table 1: Participant characteristics per subgroup of the Repeated Vaccination study.
